# Editorial: Models to study malaria parasite-host interactions and pathogenesis

**DOI:** 10.3389/fcimb.2022.1039887

**Published:** 2022-09-22

**Authors:** Maria Bernabeu, Andrea L. Conroy, Alister G. Craig, Laurent Renia

**Affiliations:** ^1^ European Molecular Biology Laboratory (EMBL) Barcelona, Barcelona, Spain; ^2^ Ryan White Center for Paediatric Infectious Diseases and Global Health, Indiana University School of Medicine, Indianapolis, IN, United States; ^3^ Liverpool School of Tropical Medicine, Liverpool, United Kingdom; ^4^ Lee Kong Chian School of Medicine, Nanyang Technological University, Singapore, Singapore

**Keywords:** malaria, model, pathogenesis, host - pathogen interactions, parasite biology

In 1976 George Box, a British statistician, wrote “All models are wrong, some are useful” and malaria models are no exception. At times passions have run high over the rightfulness or otherwise of these models (Craig et al., 2012), but our ability to translate basic knowledge into mechanistic insights into malaria pathology depends to a large extent on models. The pathologies of malaria disease, in particular severe, life-threatening clinical syndromes are defined by the nature of the combination of the invading pathogen and the host environment that the pathogen encounters and subsequently modifies. Key areas that have been identified that influence disease processes include immunity, inflammation and cytoadherence, all of which can be represented to varying extents using existing models.

Indeed, our knowledge of the biology of the malaria parasite *Plasmodium* spp. has increased dramatically since the early 1970s through improvements in molecular and cellular biology technology and the use of models. However, translating this knowledge into an understanding of malaria pathology has been more of a challenge, not least because of the need to include the context of the host-parasite relationship in these studies. This Research Topic contains papers showing how models can be used to elucidate the complex relationships between host and parasite and to dissect the molecular mechanisms that underpin these processes.

Taken together, the Research Topic highlights several considerations of models:

## Accessing the specific parasite-host material

Sourcing appropriate material from human infection is quite often impractical, and especially difficult is the obtention of tissue samples. Thus, Gupta and Wassmer review the use of miRNA as biomarkers of infection, host-pathogen interactions or severe disease. Other papers within this topic use animal and *in vitro* models as an alternative. Lyons et al used *in vitro* expression systems to produce proteins for functional and structural investigations, as well as generating antibodies as tools for research. Giorgalli et al. needed parasites from multiple stages of the rodent malaria parasites, *P. chabaudi chabaudi* to understand the complex expression patterns of PIR proteins throughout its lifecycle, and Glennon et al. faced a common issue of being able to focus on specific regions of interest of a tissue to remove the ‘noise’ from large areas of uninvolved cells, in this case to look at only the infected hepatocytes to understand the role of signalling in infection.

## Matching the model to the study

One criticism of malaria models was that the model had become the whole story, rather than a tool to understand human disease (White et al., 2010). It does not seem unreasonable to investigate the fascinating biology of parasites in their hosts, however, if this research is done to understand human disease, then validating the model (or hypothesis generated) through comparison with clinical data is warranted. The most effective experimental animal models reproduce clinical findings to answer critical questions about disease pathogenesis. Comparison of clinical features is a good starting point, as seen with Possemiers et al. and their work on malaria-induced acute kidney injury. However, this can be taken further through an even deeper analysis of wider features of different models and their relationship to different aspects of human disease. Nguee et al. demonstrate this approach well in their review on models for acute respiratory syndrome, as does the work described by Rosa-Gonçalves et al, which through careful phenotypic evaluation, allows the complex clinical phenotype of neurocognitive deficits to be investigated.

## Models can be simple or very complicated

Some models for research can be relatively simple, such as the primary human endothelial cells used by Ortolan et al. to show that specific PfEMP1 types can mediate cytoadherence to a range of tissues, which may indicate how multi-organ involvement takes place in severe malaria. However, even within cytoadherence research, the complexity of the model can step up significantly, such as the use of non-human primates (NHP) in revealing the role of SICA in *Plasmodium knowlesi* in mediating binding (Peterson et al). Recent advances in tissue engineering are increasing the complexity of *in vitro* models, including the development of 3D-microfluidic devices (Bernabeu et al., 2021) and organoids (Adams and Jensen, 2022), or stem cell approaches. For example, the extensive work done in developing cellular and *in vivo* models of erythropoiesis that have been adapted for malaria research on the effect of infection on anaemia and the behaviour of gametocytes in the bone marrow (Feldman and Egan). One of the issues of complicated models, either due to challenges in maintaining NHP appropriately or through the sheer complexity of the platform, is how to promote access across the research community.

The papers in this Research Topic show very clearly how the models that they employ can be used effectively to increase our knowledge about the biology of the host-parasite relationship and the pathology of disease. As we think about how to advance models to delineate pathways of disease in malaria, several themes emerge. First, there needs to be industry and government support so that scientists are able to establish, improve or modify existing models to adapt to the changing epidemiology of malaria and address changing research priorities. Models should be guided by clinical relevance and adapted to technology advancements and the needs of the research community over time. Partnerships should be guided by an ethical framework that supports and listens to the research priorities of scientists working in malaria endemic areas—and communities affected by malaria (Morton et al., 2022). Finally, there is a need for increased access to data sharing, common protocols, training platforms and open access publications to facilitate equitable access to the science.

**Key issues**

Models are really useful – they can elucidate complex biology, reveal the key pathological events in severe disease and down-select or validate potential therapies. So, what are some of the issues for the future?

- Supporting the development of refined or new models is important, but often not seen as innovative or moving the field forward. Supporting the development of new and better models needs investment and recognition.
- How do we support researchers who do not have access to clinical research sites so that they can test the hypotheses generated in their model systems? How do we make these partnerships equitable, including the development of the initial research questions?
- Having a variety of models can be useful but makes harmonisation of studies challenging. Can standardised models be implemented without stifling innovation and by sharing investment and access?

## Author contributions

All authors acted as editors for manuscripts submitted for the Research Topic and contributed to the writing and revision of this editorial.

## Acknowledgments

We would like to thank the authors who contributed their manuscripts to this Research Topic and who continue to support models in their research.

## Conflict of interest

The authors declare that the research was conducted in the absence of any commercial or financial relationships that could be construed as a potential conflict of interest.

## Publisher’s note

All claims expressed in this article are solely those of the authors and do not necessarily represent those of their affiliated organizations, or those of the publisher, the editors and the reviewers. Any product that may be evaluated in this article, or claim that may be made by its manufacturer, is not guaranteed or endorsed by the publisher.
